# Beyond challenging behaviors, sleep maintenance problems in autistic youth at the time of hospitalization are associated with increased caregiver strain

**DOI:** 10.1016/j.sleep.2025.108695

**Published:** 2025-12-03

**Authors:** Briana J. Taylor, Kahsi A. Pedersen, Erin R. Riddell, Yasmin Andalib, Brooklyn Tory, Matthew Siegel

**Affiliations:** a The Roux Institute at Northeastern University, 100 Fore St, Portland, ME, 04101, USA; b Department of Psychology, Northeastern University, 105-107 Forsyth St, Boston, MA, 02115, USA; c MaineHealth Institute for Research, 81 Research Drive, Scarborough, ME, 04074, USA; d Department of Psychiatry, Tufts University School of Medicine, 136 Harrison Ave, Boston, MA, 02111, USA; e Department of Psychology, McGill University, 2001 McGill College Ave, Montreal, Quebec, H3A 1G1, Canada; f Department of Psychiatry & Behavioral Sciences, Boston Children’s Hospital, 300 Longwood Ave, Boston, MA, 02115, USA

**Keywords:** Autism, Sleep problems, Parenting stress, Marital status, Socioeconomic status

## Abstract

**Purpose:**

Autistic children are at increased risk for psychiatric hospitalization for challenging behaviors. Sleep problems may increase this risk due to the added strain they place on caregivers and their ability to maintain safety at home. We evaluate if caregiver-reported sleep problems in autistic children at the time of the child’s hospital admission predict caregiver reported stress and self-efficacy, above and beyond the severity of the child’s challenging behaviors.

**Methods:**

Participants included 598 autistic children (ages 4–20) admitted to specialized psychiatric inpatient units and a primary caregiver. Caregivers reported if their child had sleep initiation and/or sleep maintenance problems. Caregivers completed the Parenting Stress Index-Short Form and Difficult Behavior Self-Efficacy Scale. Hierarchical linear regression models examined associations between sleep problems and caregiver outcomes, controlling for child irritability, and both household- and child-level characteristics.

**Results:**

Fifty-nine percent of children had a caregiver-reported sleep problem. Sleep maintenance problems were significantly associated with increased parental distress, more dysfunctional parent-child interactions, and greater perception of the child as difficult to manage, above and beyond challenging behavior severity and other covariates. Sleep initiation problems were unrelated to caregiver outcomes. Self-efficacy in single caregivers and those in lower-income households was more negatively impacted by sleep problems than other caregivers.

**Conclusions:**

Sleep maintenance problems may independently contribute to caregiver strain above and beyond challenging behaviors. Social and financial resources may buffer against the negative effect of sleep problems. Preventative interventions targeting sleep may provide needed support for vulnerable families.

Autistic youth are hospitalized for psychiatric reasons at approximately six times the rate of their non-autistic peers, often due to acute behavioral crises such as aggression or self-injury that pose safety risks [1,2]. These crises typically emerge from complex, chronic stressors that unfold within the family system rather than from sudden or isolated events. One understudied factor that may contribute to crisis escalation is sleep disturbance. Despite growing recognition that sleep problems are both prevalent and persistent in autistic youth, their role in psychiatric hospitalization risk and caregiver functioning has received little empirical attention.

Up to 80 % of autistic youth experience at least one significant sleep problem, with the most common being sleep initiation problems (i.e., difficulty falling asleep) and sleep maintenance problems (i.e., difficulty staying asleep) [3]. Sleep problems are highly interconnected with daytime functioning; they can exacerbate challenging daytime behaviors, and these behaviors in turn can further disrupt sleep, creating escalating bidirectional cycles over time [4]. Unlike daytime challenging behavior, however, sleep-related problems occur at night when behavioral health supports are rarely available, and caregivers are typically managing these sleep issues alone. Nighttime safety risks are amplified when behaviors such as self-injury or elopement occur during nocturnal awakenings, when caregiver supervision is naturally reduced.

These chronic sleep disturbances can convert caregiving into a 24-h unrelenting responsibility with an eroding effect on caregivers’ ability to maintain their child’s safety at home [5,6]. Although the relationship between child sleep problems and caregiver stress is well established, little is known about these dynamics in families facing psychiatric crises severe enough to require inpatient hospitalization. The current study evaluates whether sleep problems in autistic youth are associated with variance in caregiver stress and self-efficacy above and beyond the most cited behavioral reasons for hospitalization (aggression and self-injury). Additionally, we explore the moderating effects of household characteristics (caregiver marital status and household income) that may relate to nighttime and daytime access to support, and child characteristics (age and functioning level) that may relate to the degree of caregiver involvement required during sleep disruptions. Studying sleep and caregiver functioning in this high-acuity context offers a unique opportunity to understand factors that may contribute to crisis escalation and inform preventative support tools.

Caregivers of autistic youth consistently report higher levels of stress compared to caregivers of non-autistic youth [7] and those of youth with other disabilities [8–10]. Caregiving stress is often linked to managing challenging daytime behaviors [11], but is also associated with the difficult and exhausting responsibility of finding and coordinating support services, specialized healthcare providers, and appropriate educational programming [12,13]. Chronic sleep problems compound these challenges by disrupting caregivers’ own sleep and capacity to manage both challenging behaviors and the increased demands of caring for an autistic youth. Overtime, this cycle can have an eroding effect on caregiver self-efficacy, the sense of confidence in one’s ability to maintain safety and support a child’s needs, creating a feedback loop of heightened stress and vulnerability to crisis [14,15].

Resources, both social and financial, may moderate the extent to which sleep problems in autistic youth affect caregiving stress and self-efficacy. Perceived support from partners, family members, and friends have predicted less stress, better well-being, and greater use of positive coping strategies in caregivers of autistic youth [16–18]. The marital relationship in particular has been associated with better quality of life for families with autistic youth [19]. Support from spouses or live-in partners may be particularly important in the context of sleep problems given their ability to provide tangible, real-time support at the time of awakenings [20]. Of course, this hypothesis relies on the assumption that partners share the job of sleep problem management, and undoubtedly numerous sociocultural factors influence the distribution of caregiver responsibilities in couples. Nevertheless, more than one caregiver within the home presents the opportunity for co-management and may buffer against the detrimental impact of sleep problems in youth on caregiver stress and self-efficacy.

Financial resources may also buffer the impact of sleep problems in youth on caregiver functioning. Caregivers of autistic youth often experience increased financial burden [21,22]. The additional cost of raising an autistic youth is estimated to be between US$3020 - US$5600 per year in healthcare expenditures and over US$14,000 per year in non-healthcare costs [23]. Higher household income has been associated with reduced mental health problems in caregivers of autistic youth and better perceived quality of life for families [19,24]. Moreover, lower household income has been associated with increased sleep problems in autistic youth [25] and chronic sleep problems in caregivers may limit their workforce participation. Alternatively, greater socioeconomic resources may allow families to access overnight respite care or find employment with flexible work schedules to accommodate the shifting needs of struggling caregivers. Those with higher household incomes may have greater access to healthcare specialists such as those with training in sleep medicine. Finally, higher household income may reflect greater educational attainment which may enable caregivers to better consume information about evidence-based sleep hygiene recommendations and navigate the healthcare landscape more effectively [26].

The potential burden of sleep problems may also vary by autism phenotypes and co-occurring conditions. Autism is highly heterogenous, yet many studies overlook key individual differences such as variations in autism symptom severity, verbal ability, and intellectual functioning. Autism symptom severity may modulate the extent to which sleep problems contribute to caregiver stress. Those with profound autism, characterized by the need for 24/7 access to an adult, minimal verbal ability or co-occurring intellectual disability, often have needs that demand more intensive involvement from caregivers. Research shows that caregivers of youth with profound autism have reported more stress, less resilience, and lower self-efficacy; however, research regarding sleep problems in this population is limited and mixed [27–30]. Understanding how sleep problems relate to caregiver stress and self-efficacy across different autism phenotypes and co-occurring conditions can help identify the most vulnerable families and inform the development of more personalized, family-based sleep interventions.

Finally, sleep problems and their nature vary with age due to developmental shifts in the circadian and homeostatic mechanisms underlying sleep [31]. The transition into adolescence is associated with shifts toward a “night owl chronotype”, characterized by later sleep and wake times and, due to early school start times, shorter sleep duration and increased daytime sleepiness. Autistic adolescents also exhibit a night owl chronotype, which has been identified in the broader literature as a transdiagnostic risk factor for mental and behavioral health problems [32,33]. Older autistic youth also show decreased sleep initiation and maintenance problems than younger youth and may require less hands-on support and/or supervision from caregivers [32]. Despite the known impact of age on sleep, it is unclear how age-related shifts in sleep problems impact caregiver stress and self-efficacy, and ultimately the extent to which sleep problems may erode a family’s ability to remain intact without crisis-level intervention.

The current study evaluates if sleep problems in autistic youth contribute to the variances in caregiver stress and self-efficacy above and beyond challenging behaviors. Additionally, we explore the moderating effect of household characteristics (i.e., caregiver marital status and household income) and child characteristics (i.e., age, the presence of profound autism, and level of irritability). Moderation analyses will clarify the context in which sleep problems may impact caregiver stress and self-efficacy, which are key mechanisms underlying caregivers’ ability to safely manage challenging behaviors within the home. Because these data are drawn from families seeking inpatient psychiatric care, findings should be interpreted within the context of this high-acuity population and not generalized to all families of autistic youth. Rather, they inform clinical practice for professionals working with families at risk for, or recovering from, psychiatric crises.

We hypothesize that (1) sleep problems in autistic youth will be associated with increased caregiver stress and decreased self-efficacy; (2) sleep maintenance problems, alone or in combination with sleep initiation problems, will have the strongest association with caregiver stress and self-efficacy; and (3) the impact of sleep problems on caregiver stress and self-efficacy will be greater for families with fewer social and financial resources. Given the limited and mixed background literature, moderation analyses by child characteristics are exploratory in nature.

## 1. Methods

### 1.1. Participants

Participants in this study included 598 autistic youth who were admitted to a pediatric inpatient psychiatry unit. Participants were 4–20 years old and their primary caregiver. Participants were recruited through a larger study, the Autism Inpatient Collection (AIC) [34], a multisite study of youth admitted to one of six psychiatric inpatient units specialized for the treatment of autistic youth and those with other neurodevelopmental conditions. The study was approved by the Institutional Review Board for each site of the AIC. Primary caregivers present at the time of admission were given the opportunity to participate in the research study and, if they opted to do so, provided written informed consent at that time or within five days of admission. Due to the high prevalence of profound autism within the sample, characterized by limited verbal ability and/or co-occurring intellectual disability, assent was not sought directly from autistic youth.

### 1.2. Procedure

Following informed consent, one primary caregiver was asked to complete a set of questionnaires. When more than one primary caregiver was present, caregivers decided amongst themselves who would complete the questionnaires. Caregivers were given the option to complete the questionnaires at the time of their child’s admission or at home within 10 days of the admission date. During the hospital stay, diagnosis of autism spectrum disorder was made by an examiner who was research reliable on the Autism Diagnostic Observation Schedule, 2nd Edition (ADOS-2), and each participant’s diagnosis was supported by empirically derived cutoff scores on the ADOS-2 and meeting DSM-5 criteria for ASD. Non-verbal IQ was assessed by the Leiter International Performance Scale instrument. The ADOS-2 and non-verbal IQ assessment were completed by trained and research-reliable research staff members who were either clinical psychologists or clinical research coordinators. For further details on the AIC protocol, please see Siegel et al. (2015).

### 1.3. Caregiver-report measures

#### Demographic Information –

Caregivers provided demographic information including the youth’s age, sex, race, ethnicity, their own relationship to the autistic youth (e.g., biological parent, stepparent, etc.), household income (U.S. dollars), and the primary caregiver’s marital status. Caregivers reported on their youth’s race and ethnicity, with the latter defined by Hispanic or non-Hispanic heritage. Caregivers indicated their household income by selecting one of 9 brackets ranging from less than US$10,000 to greater than US$120,000. Household income was operationalized dichotomously as ≤ US$50,000 per year or > US$50,000 per year, as approximately US$50,000 per year reflected the median income in the sample. For reference, the median household income in the U.S. ranged from approximately US$54,000 to US$63,000 during the period of data collection for this phase of the AIC (2014–2018). Caregivers indicated their marital status using one of 4 designations: married, single, widowed, living with a partner. To differentiate caregivers who did and did not have a partner within the home, those who were married or living with a partner were combined in one group, labeled “married”, and those who were single or widowed were combined in another group, labeled “single”. Given the wide age-range of the population and known changes in sleep across the transition into adolescence, we evaluated interactions by the youth’s age as a dichotomous variable (i.e., ≤12 years old and ≥13 years old).

#### Sleep Problems –

Intake forms included questions to determine the presence and nature of sleep problems. On the form, participants were asked, “Does your child have significant sleeping problems?”. If they answered “yes”, they were asked to specify and were provided the options: “difficulty falling asleep”, “difficulty staying asleep”, or “other”. Difficulties with falling and staying asleep were specified, as they reflect the most common sleep problems among autistic youth [3]. Within the “other” category, several caregivers endorsed “early morning awakening”. Because this latter problem was common but not prevalent enough to be analyzed separately in moderation analyses due to small cell sizes, “early morning awakenings” and “difficulty staying asleep” were collapsed into one “sleep maintenance problem” category. Difficulty falling asleep was re-named “sleep initiation problem” for consistent language. Finally, a significant portion of participants had both sleep initiation and sleep maintenance problems. As multiple sleep problems may confer additional burden for caregivers, this group was regarded as a separate category. Therefore, participants were grouped into four categories: no sleep problems, sleep initiation problems, sleep maintenance problems, and sleep initiation & maintenance problems (SI + SM). Nineteen caregivers reported other sleep problems including apnea or snoring, nightmares, hypersomnolence, enuresis, and sleep-walking, talking, or eating. Six caregivers who reported other sleep problems with no accompanying sleep initiation or maintenance problems were not included in one of the three sleep problem groups.

#### Parent Stress Index – Short Form (PSI-SF) [35] –

The PSI-SF is a self-report measure designed to assess stress in caregivers of children. The term “parent” is used throughout the manuscript when referring to the PSI-SF subscales and elsewhere, the more inclusive term, “caregiver” is used. The PSI-SF examines three domains of stress: parental distress, parent-child dysfunctional interaction, and difficult child characteristics. The parental distress subscale reflects the extent to which a caregiver feels conflicted or restricted in their role as a caregiver. The parent-child dysfunctional interactions subscale reflects the extent to which a caregiver feels satisfied or disappointed with their caregiver-child relationship. The difficult child subscale reflects the extent to which caregivers perceive that their child is particularly difficult to care for. The PSI has demonstrated good internal consistency (*α* = 0.80) in numerous samples [36]. Higher scores reflect more stress in each domain. The PSI-SF was originally validated for caregivers of children ages 1 month to 12 years old and thus, results presented here should be interpreted with caution as children in our sample ranged in age from 5 to 20 years old. However, it should be noted that the PSI demonstrates moderate-to-strong concordance with the Stress Index for Parents of Adolescents (SIPA) [37], a measure that was developed later as an upward extension of the PSI for caregivers of adolescents. Strong correlations between the two suggest that constructs measured by each measure are relatively similar [38].

#### Difficult Behavior Self-Efficacy Scale (DBSS) [39] –

The DBSS is a self-report questionnaire designed to measure individuals’ confidence in their ability to manage challenging or difficult behaviors in children or adolescents with developmental disabilities. It assesses perceived self-efficacy in handling various behavioral issues, such as aggression, noncompliance, or emotional dysregulation. Oh and Kozub (2010) report that the measure demonstrates high internal consistency (*α* = 0.88). Higher scores reflect greater self-efficacy.

#### Aberrant Behavior Checklist – Irritability (ABC-I) [40] –

The ABC-I subscale is designed to assess the presence and severity of irritability-related behaviors in children and adolescents with developmental disabilities, ages 5 years old and older. The ABCI-I was chosen due to its high internal consistency (*α* = 0.95) and test-retest reliability (*r* = 0.98) [41]. It focuses on behaviors that reflect emotional dysregulation, frustration, and anger, including aggression, self-injurious behavior, temper tantrums, verbal aggression, and destruction of property. The ABC-I captures the behaviors that are the primary reasons for psychiatric hospitalization of autistic youth [42]. Controlling for ABC-I scores will reveal if sleep problems in autistic youth account for variance in caregiver stress and self-efficacy above and beyond challenging behaviors.

### 1.4. Assessments completed by AIC study staff

#### Autism Diagnostic Observation Schedule, Second Edition (ADOS-2) [43] –

The ADOS-2 is a semi-structured assessment tool used to assist with the diagnosis of autism. It involves direct observation by the diagnostician of social interaction, communication, and play to evaluate the severity of autism symptoms to produce two subscale scores: social-affective symptom severity and repetitive behaviors and restricted interests. The ADOS is widely regarded as the Gold Standard for autism diagnosis, and has demonstrated high validity, with sensitivity ranging from 83 % to 91 % and specificity from 86 % to 94 % [44]. Different modules are administered depending on the age and verbal ability of the child. In the current analysis, children were assessed with Modules 1–3. Module 1 is for children 31 months and older who don’t use phrase speech. Module 2 is for children of any age who use phrase speech but aren’t verbally fluent. Module 3 is for verbally fluent children and young adolescents. An age-based comparison score is also produced and used in the current study with higher values representing greater autism symptom severity. The ADOS-2 module selected by the examiner was used as an indicator of verbal ability with modules 1 and 2 reflecting minimal verbal ability and modules 3 and 4 reflecting fluent verbal ability.

#### Leiter International Performance Scale (Leiter-3) [45] –

The Leiter-3 is a nonverbal intelligence test designed to assess cognitive abilities, particularly in individuals who may have difficulty with language or verbal expression and designed for individuals 3–75 years old. This assessment has both high validity measures (*α* = 0.88 to 0.93). It focuses on visual-spatial reasoning, attention to detail, and problem-solving skills without relying on verbal instructions or responses. The Leiter-3 produces standardized scores with a mean of 100 and scores ≤55 reflecting profound intellectual disability.

### 1.5. Statistical analysis

ANOVAs and chi-square (*X*^*2*^) tests were used to evaluate differences in child, caregiver, and household characteristics across the sleep problem groups: no sleep problems, sleep initiation problems, sleep maintenance problems, and SI + SM problems. Hierarchical linear regression models were used to examine associations between children’s sleep problems and the three subscales of PSI-SF and the DBSS measure. Interactions between sleep problems and household level characteristics (i.e., caregiver marital status and household income > US$50,000) and child level characteristics (i.e., age and profound autism) were evaluated in separate regression models. A dichotomous variable for child’s age (i.e., ≤12 years old and ≥13 years old) was used due to known changes in sleep between children and adolescents and for ease of interpreting interactions. The variable of profound autism was created and defined in this sample as having a Leiter score of ≤55 or being minimally-verbal (ADOS modules 1 or 2). This single variable was used as a covariate and moderator to preserve degrees of freedom given the limited sample size and number of tests. Analyses were adjusted for ABC-I scores and each moderator. Child sex, caregiver type (i.e., biological parent or other), and autism symptom severity were evaluated as potential covariates but were not included in the final models due to their lack of association with the outcome variables and predictors. Univariate analyses and pairwise comparisons were evaluated when significant interaction effects were found. A threshold of *α* = 0.05 was used to determine statistical significance. Effect sizes (partial *η*^*2*^) are also reported for results. For reference, partial *η*^*2*^ values reflect the following effect sizes: 0.01–0.05 = small effect, 0.06–0.13 = medium effect, 0.14–0.20 = large effect) [46].

A sensitivity analysis was conducted using a subsample of 501 participants for whom medication information was available to examine the potential influence of sleep medications on associations between sleep variables and parenting stress and self-efficacy. Sleep medication use was not included in the primary models because available data reflected medications recorded on the first night of hospitalization and may not represent home medication regimens. Although physicians rarely altered sleep-related medications during admission, potential variability in prescribing practices across the six inpatient units limits confidence in the medication data. In addition, supplemental melatonin was the only clear sleep-specific medication in our dataset. Although some participants were prescribed psychotropic medications such as guanfacine or risperidone that may have been administered in the evenings for their sedating effects, our data did not include information on the timing of administration or if these medications were intended to address sleep problems or other challenges. Therefore, our sensitivity analysis was limited to the prescription of supplemental melatonin on the participant’s first night of hospital stay.

## 2. Results

The demographic makeup of the study sample is shown in Table 1. Children had a mean age of 12.9 ± 3.5 years old and were mostly male, white, and non-Hispanic. Approximately one-fourth had co-occurring profound intellectual disability (22 %) and close to half were minimally-verbal (45 %). Most caregiver respondents were the child’s biological parent (78 %), approximately one-third were single or not living with a partner (35 %) and half of families had a household income above US$50,000 per year (54 %). More than half of the child participants (N = 350) had a caregiver-reported sleep problem (59 %). Of those with sleep problems, 90 had a sleep initiation problem (26 %), 99 had a sleep maintenance problem (28 %), and 161 had both a sleep initiation and a sleep maintenance problem (46 %).

Children with sleep maintenance problems were significantly older than those with no sleep problems (mean difference = 0.8 years, standard error = 0.41, *p* = 0.05), sleep initiation problems (mean difference = 1.2 years, standard error = 0.51, *p* = 0.02), and SI + SM problems (mean difference = 1.3 years, standard error = 0.44, *p* = 0.003). Children with sleep maintenance problems also had significantly lower non-verbal IQs than those with no sleep problems (mean difference = −10.8 points, standard error = 3.63, *p* = 0.003), sleep initiation problems (mean difference = −9.7 points, standard error = 4.49, *p* = 0.03), and SI + SM problems (mean difference = −8.0 points, standard error = 3.95, *p* = 0.04). Children with sleep maintenance problems and children with SI + SM problems were more likely to be minimally verbal relative to children with no sleep problems (*X*^*2*^ = 4.63, *p* = 0.03 and *X*^*2*^ = 8.09, *p* = 0.004, respectively).

### 2.1. Main effects of child sleep problems on caregiver stress and self- efficacy

There were no main effects of sleep initiation problems on caregiver outcomes (all *p*-values >0.05, see Table 2). In fully adjusted models, sleep maintenance problems were associated with higher parental distress (*β* = 0.09, *p* = 0.05, partial *η*^*2*^ = 0.01), greater parent-child dysfunctional interactions (*β* = 0.13, *p* = 0.002, partial *η*^*2*^ = 0.01), and greater perception of the child as difficult (*β* = 0.12, *p* = 0.002, partial *η*^*2*^ = 0.01). SI + SM problems were associated with higher parental distress (*β* = 0.10, *p* = 0.03, partial *η*^*2*^ = 0.01), and greater perception of the child as difficult (*β* = 0.15, *p* < 0.001, partial *η*^*2*^ = 0.02). There were no main effects of any sleep problem on caregivers’ self-efficacy in managing difficult behaviors (all *p*-values >0.05).

### 2.2. Moderation by caregiver marital status

**Fig. 1.**
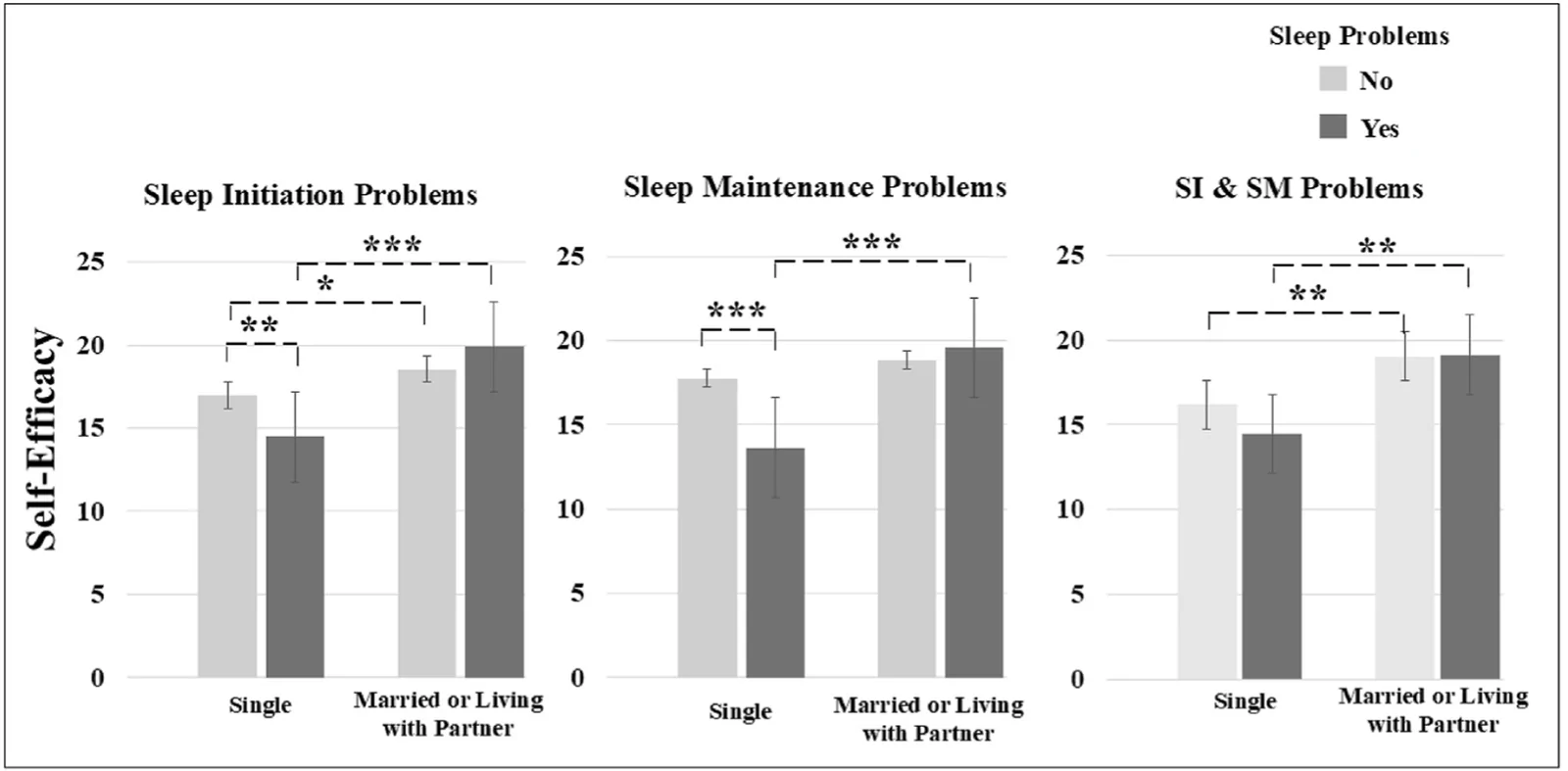
Caregiver marital status moderates associations between children’s sleep problems and parental self-efficacy.

There were no main effects of caregiver marital status on caregiver stress or self-efficacy in managing difficult behaviors. Caregiver marital status did not moderate the effect of any sleep problem on any of the three PSI subscales. However, caregiver marital status moderated the effects of sleep initiation problems and sleep maintenance problems on self-efficacy (*β* = 0.18, *p* = 0.01, partial *η*^*2*^ = 0.01 and *β* = 0.25, *p* < 0.001, partial *η*^*2*^ = 0.02, respectively). Pairwise comparisons (Fig. 1) revealed that for caregivers of children with and without sleep initiation problems, those who were married or living with a partner reported greater self-efficacy than single caregivers. However, the effect of marital status was greater for caregivers of children with sleep initiation problems (*F*(1, 586) = 10.62, *p* = 0.001, partial *η*^*2*^ = 0.02) relative to those of children without (*F*(1, 586) = 4.90, *p* = 0.03, partial *η*^*2*^ = 0.01). Single caregivers of children with sleep initiation problems also reported lower self-efficacy than single caregivers of children without sleep initiation problems (*F*(1, 586) = 7.29, *p* = 0.007, partial *η*^*2*^ = 0.01).

Similarly, for caregivers of children with sleep maintenance problems, those who were married or living with a partner reported greater self-efficacy than those who were single (*F*(1, 586) = 12.75, *p* < 0.001, partial *η*^*2*^ = 0.02) and single caregivers of children with sleep maintenance problems reported lower self-efficacy than single caregivers of children without sleep maintenance problems (*F*(1, 586) = 12.66, *p* < 0.001, partial *η*^*2*^ = 0.02). Caregivers who were married or living with a partner reported significantly greater self-efficacy than single caregivers regardless of SI + SM problems.

### 2.3. Moderation by household income

**Fig. 2.**
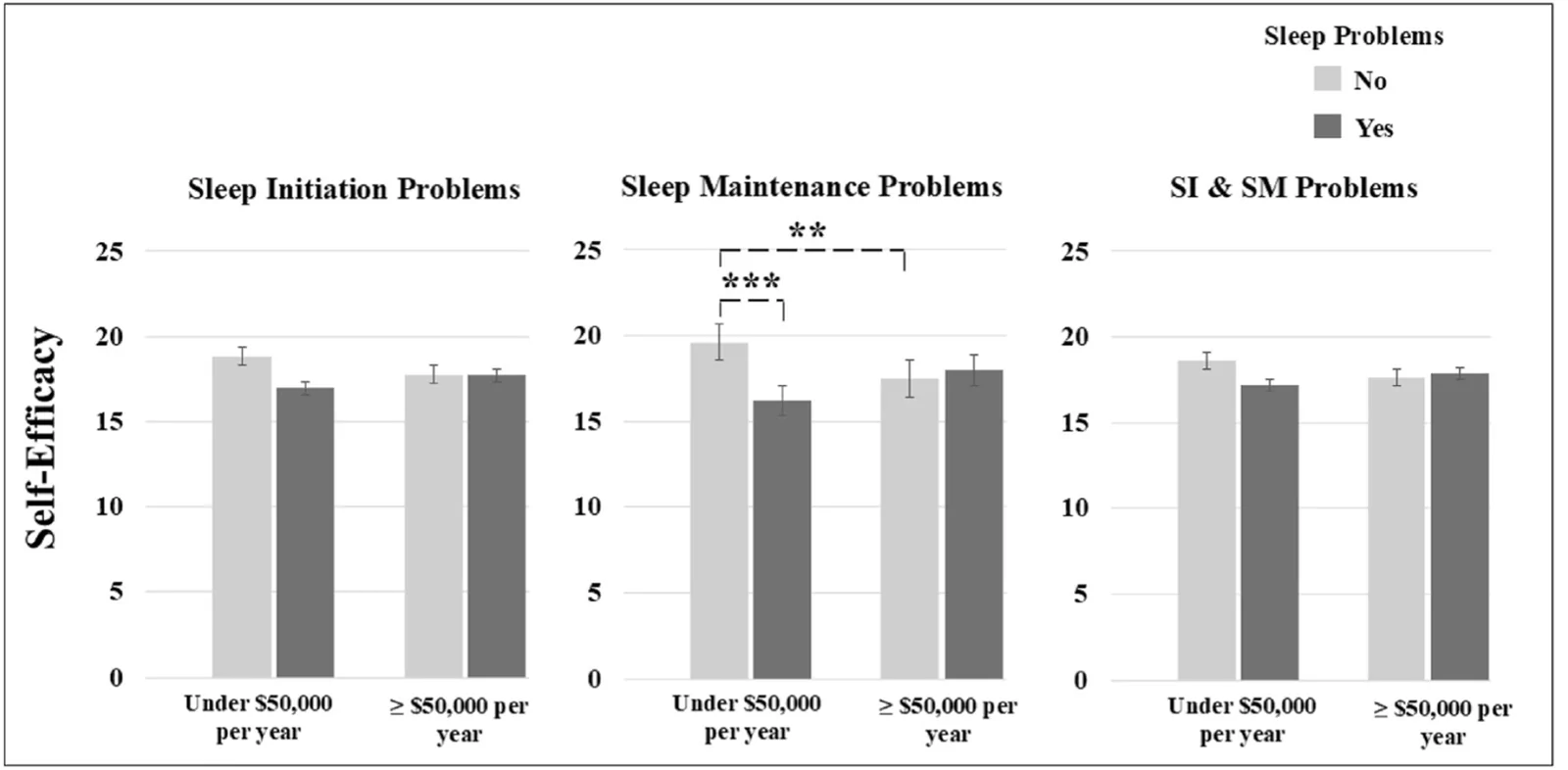
Household income moderates associations between sleep maintenance problems and parental self-efficacy.

Household income did not moderate the effect of any sleep problem on any of the three subscales of the PSI-SF but did moderate the effect of sleep maintenance problems on the difficult behavior self-efficacy scale (*β* = 0.19, *p* = 0.005, partial *η*^*2*^ = 0.01). Pairwise comparisons (Fig. 2) revealed that caregivers with annual household incomes < US$50,000 reported significantly lower self-efficacy if they had a child with sleep maintenance problems than if they did not (*F*(1, 586) = 10.34, *p* = 0.001, partial *η*^*2*^ = 0.02). Caregivers in lower income households also reported higher self-efficacy than those in higher income households but only in the absence of sleep maintenance problems (*F*(1, 586) = 6.49, *p* = 0.01, partial *η*^*2*^ = 0.01).

### 2.4. Moderation by child characteristics

Neither age nor profound autism, as defined by having co-occurring profound intellectual disability or being minimally verbal, moderated the effects of sleep problems on any caregiver outcome.

### 2.5. Sensitivity analyses controlling for supplemental melatonin

In the 501 participants with medication information, N = 140 (27.9 %) were prescribed supplemental melatonin on the first night of their hospital admission. Of this group, 19.3 % had sleep initiation problems, 16.4 % had sleep maintenance problems and 30 % had SI + SM problems. Interestingly, the largest percentage of those taking melatonin (N = 48, 34.3 %) had no parent-reported sleep problems. However, from these data, it is unclear whether the absence of sleep problems reflects effective management with supplemental melatonin, underreporting of sleep problems by caregivers, or unnecessary/preventative use of melatonin.

The main effects of sleep on parent stress and self-efficacy were largely unchanged when supplemental melatonin use was included as a covariate in the statistical models. Sleep initiation problems were unrelated to parent outcomes. Sleep maintenance problems were no longer associated with greater parental distress (*β* = 0.09, *p* = 0.06, partial *η*^*2*^ = 0.01) but were associated with greater parent-child dysfunctional interactions (*β* = 0.16, *p* < 0.001, partial *η*^*2*^ = 0.01) and greater perception of the child as difficult (*β* = 0.15, *p* < 0.001, partial *η*^*2*^ = 0.02). SI + SM problems were associated with greater parental distress (*β* = 0.10, *p* = 0.03, partial *η*^*2*^ = 0.01) and greater perception of the child as difficult (*β* = 0.18, *p* < 0.001, partial *η*^*2*^ = 0.03).

Interaction patterns were slightly impacted when controlling for supplemental melatonin, such that there was no longer a significant interaction between marital status and sleep initiation problems. Marital status continued to moderate the association between sleep maintenance issues and self-efficacy (*β* = 0.22, *p* = 0.01, partial *η*^*2*^ = 0.01). Sleep maintenance problems were associated with lower self-efficacy for single caregivers (*F*(1, 488) = 8.09, *p* = 0.005, partial *η*^*2*^ = 0.02) and married caregivers reported greater self-efficacy than single caregivers when they had children with sleep maintenance problems (*F*(1, 488) = 7.83, *p* = 0.005, partial *η*^*2*^ = 0.02).

Household income continued to moderate the association between sleep maintenance and self-efficacy (*β* = 0.18, *p* = 0.02, partial *η*^*2*^ = 0.01). Caregivers with lower household incomes reported greater self-efficacy if they had a child without sleep maintenance issues relative to those with sleep maintenance issues (*F*(1, 488) = 7.65, *p* = 0.006, partial *η*^*2*^ = 0.02). Self-efficacy no longer varied by high and low household income for caregivers of children without sleep maintenance problems (*F*(1, 488) = 3.01, *p* = 0.08, partial *η*^*2*^ = 0.01).

When supplemental medication was controlled for, there was a significant interaction between child age and sleep initiation problems on caregiver self-efficacy (*β* = 0.16, *p* = 0.03, partial *η*^*2*^ = 0.01). Sleep initiation problems were associated with lower self-efficacy for caregivers of older autistic youth ≥12 years old (*F*(1, 488) = 5.13, *p* = 0.02, partial *η*^*2*^ = 0.01) but not younger and self-efficacy was lower for caregivers of older relative to younger autistic youth when sleep initiation problems were present (*F*(1, 488) = 4.09, *p* = 0.04, partial *η*^*2*^ = 0.01).

## 3. Discussion

This study identifies child sleep maintenance problems as a distinct correlate of caregiver stress in families of autistic youth admitted for psychiatric hospitalization, above and beyond the variance accounted for by challenging behaviors. These findings highlight the importance of identifying and addressing sleep maintenance difficulties in autistic youth with high behavioral acuity, as these issues may exacerbate family stress and reduce caregivers’ capacity for managing the challenging behaviors that precipitate hospitalization. These findings build upon a large body of research regarding the detrimental nature of sleep problems in autistic youth and extend this evidence to those requiring inpatient psychiatric care. In our inpatient sample, the rate of caregivers reporting sleep problems in their child (59 %) is somewhat lower than the 70–80 % prevalence typically reported in clinical samples of autistic youth [3,47]. In the present context, acute behavioral or safety concerns may have been most salient to caregivers at the time of admission and data collection, potentially overshadowing sleep problems. Thus, the proportion of children reported to have no sleep problems (41 %) may underestimate the true prevalence of sleep difficulties in the high behavioral acuity population.

The current study sheds light on the particular significance of sleep maintenance problems (i.e., frequent nighttime awakenings and early morning awakenings) which tend to be less responsive to standard treatments such as sleep hygiene education, behavioral and environmental changes, psychotropic medications, and chronotherapeutic interventions [48–51]. Lack of effective treatment options may increase caregiver stress and reduce caregivers’ perceived ability to control sleep problems over time. Reduced perceived control over sleep problem management has been associated with reduced adherence to behavioral sleep medicine recommendations, which may perpetuate sleep problems in children and caregivers long-term [52]. In caregivers of autistic youth, sleep problems are common [53–56] and are associated with increased caregiver burden, reduced quality of life, and more mental health problems [57–59]. Caregivers of autistic youth report that their own exhaustion is a major barrier to implementing behavioral interventions for their children [60,61], suggesting that feedback and feedforward loops between sleep and daytime behavior are likely to interact with the degree to which caregiver functioning is impacted. Whereas child sleep initiation problems may delay a caregiver’s sleep, sleep maintenance problems can result in repeated interruptions which make it difficult for caregivers to complete full sleep cycles [62,63], with marked consequences emotion regulation and reactivity to stress [64–66].

### 3.1. Sociodemographic vulnerabilities

The moderating effects of marital status suggest that single caregivers may be more vulnerable to the negative impact of their child’s sleep problems, suggesting that sharing caregiver responsibilities and a support system may buffer against the detrimental effects of sleep problems. This finding aligns with theories of dyadic coping, where couples can engage in collaborative problem-solving and mutual support when facing stressors [67]. Consistent with this interpretation, Righi et al. found that both sleep problems and living in a single-parent household independently increased the likelihood of psychiatric hospitalization among autistic youth, underscoring the combined strain of sleep disruption and limited caregiving support [2]. Community-based interventions for families of autistic children with high behavioral acuity and sleep maintenance problems may consider how additional support could be provided to single-caregivers who are managing 24-h caregiving alone. For instance, Mandell et al. demonstrated that access to community-based supports such as respite care significantly reduced the demand for inpatient psychiatric hospitalization among autistic children [68], highlighting the potential value of interventions that extend caregiving resources beyond the immediate family.

Household income also emerged as a critical moderator, such that lower-income caregivers reported significantly lower self-efficacy if their child experienced sleep maintenance problems, while higher-income families showed no such relationship. This finding is consistent with those in caregivers of non-autistic youth, reporting that child sleep problems are perceived as significantly more taxing when resources are already strained [69,70] and are correlated with lower caregiver self-efficacy for those in low-income households [71,72]. Financial resources may enable caregivers to access overnight respite care, increased daytime support, or mental health resources that help caregivers to cope with persistent sleep disruption. The current report reinforces the need to consider socioeconomic status when evaluating and treating sleep problems in autistic youth, and the need for additional support for families with fewer resources.

### 3.2. Supplemental melatonin

Sensitivity analyses controlling for supplemental melatonin revealed that the main effects of child sleep problems on caregiver stress and self-efficacy were largely unchanged, suggesting that the associations observed were not primarily driven by melatonin use. However, findings also highlight the complexity of interpreting melatonin use in this population. A substantial portion of children prescribed melatonin had no parent-reported sleep problems, indicating that melatonin is effectively treating sleep issues for a portion of participants. Alternatively, results may also suggest that melatonin is being used by some prophylactically. The use of melatonin in the absence of significant sleep problems cannot be ruled out, particularly given its growing popularity and perception within the community as a benign, “vitamin-like” supplement rather than a synthetic hormone with limited evidence regarding long-term safety [73,74]. Misinformation about melatonin’s role in sleep further complicates its use; melatonin acts primarily as a circadian phase signal rather than a soporific agent and may have limited utility for sleep maintenance or early-morning awakenings. Future studies are needed to characterize which subgroups of autistic youth respond to pharmacological versus behavioral sleep interventions and to identify the factors that guide caregiver and clinical decision-making around melatonin use. A more precise understanding of treatment response profiles will help optimize sleep management strategies, reduce unnecessary pharmacologic exposure, and better support families struggling with chronic sleep disruption.

### 3.3. Clinical implications during admission

Sleep maintenance problems may represent an important modifiable intervention target during hospitalization. Inpatient admission offers a controlled environment in which sleep patterns can be observed and stabilized while caregivers receive psychoeducation and support. Incorporating sleep screening and management into inpatient care plans could reduce caregiver stress and improve post-discharge outcomes. At discharge, families could receive specific follow-up plans for addressing sleep, comparable to those provided for behavior management or medication monitoring. Outpatient follow-up with behavioral sleep specialists or telehealth-based parent coaching could support continuity of care. For caregivers with limited social or financial resources, linkage to community-based respite programs or family navigation services may buffer stress and enhance adherence to sleep interventions.

### 3.4. Tertiary prevention for recurrently admitted youth

For the subset of autistic youth with repeated hospitalizations, sleep problems may signal an ongoing and unaddressed driver of family system breakdown. Integrating sleep-focused secondary and tertiary prevention efforts into outpatient or wraparound services could reduce recurrence. Examples include longitudinal monitoring of sleep quality as part of care coordination, routine screening for sleep disturbance during outpatient visits, and embedding behavioral sleep intervention within outpatient or home-based services. Because single caregivers and low-income families in this study reported the lowest self-efficacy when managing sleep maintenance problems, these preventive efforts should explicitly account for family structure and socioeconomic context, such as offering flexible scheduling, caregiver coaching, or in-home support when available.

### 3.5. Strengths, limitations, and future directions

The present study extends the literature by demonstrating associations between pediatric sleep problems and caregiver strain within families of autistic youth with high behavioral acuity, requiring inpatient care. Understanding modifiable contributions to caregiver stress and self-efficacy is essential to develop preventative interventions that could ultimately reduce the need for crisis service utilization, such as hospitalization. As these data were from the Autism Inpatient Collection, the current analysis serves as an important and unique view into family dynamics at the critical time of accessing a crisis-level intervention. In addition, the current study advances our understanding of the contexts within which children’s sleep problems increase risk for families. Specifically, we evaluated the moderating role of sociodemographic household-level factors which are rarely considered when developing preventative interventions. The current sample is enriched for lower socioeconomic status families and single-caregiver households who face unique struggles when implementing and adhering to behavioral interventions and are underrepresented in autism research [75].

The current study is also enriched for youth who can be categorized as having “profound autism” (e.g., having co-occurring intellectual disability and/or being minimally verbal). Research shows that individuals with profound autism have been increasingly excluded from clinical research since the 1990s [76]. Increased representation of youth with profound autism in research is essential as sleep problems and other behavioral challenges may differ in presentation, treatment needs, and the level of support that caregivers need to manage them effectively.

The results of this study must also be considered within the lens of design limitations. Due to the cross-sectional nature of these data, we cannot establish causal relationships between child sleep problems, caregiver stress and self-efficacy. Longitudinal studies are needed to understand the chronology of child sleep problems, caregiver stress, and caregiver self-efficacy. Moreover, studies that measure child and household characteristics prior to hospital admission can help us to understand how family dynamics contribute to the need for hospitalization and identify appropriate community-based services. In addition, information on the specific reason for admission was not available in our data and could not be examined as a potential moderator. Finally, medication data were limited to prescriptions recorded on the first night of hospitalization, which may not reflect home use or prescribing intent. Because of these methodological constraints and variability across sites, sleep medication use was excluded from the primary analyses. Future studies should capture outpatient medication histories, dosing, and indication to better characterize pharmacologic management of sleep problems in autistic youth.

The use of the PSI-SF as the primary measure of parent stress is also something to consider when interpreting the results of this study. As mentioned in the methods section, the PSI is validated for caregivers of children up to 12 years old; however, to simplify the process of collecting data through a multi-site study, we administered the PSI-SF to all caregivers of autistic youth admitted to the specialized pediatric inpatient units who could be up to 20 years of age. Despite this potential limitation, the PSI has shown high convergent validity with the Stress Index for Parents of Adolescents (SIPA) [37,38], a measure designed as an upward extension of the PSI. Given the demonstrated continuity between the PSI and SIPA, the PSI may have served as an imperfect but acceptable measure of caregiver stress for a broad pediatric sample.

## 4. Conclusions

The current study extends the literature on sleep problems in autistic youth by demonstrating that sleep problems are associated with greater caregiver stress above and beyond the severity of challenging behaviors in an inpatient sample with high behavioral acuity. These findings suggest that clinicians working with autistic youth in inpatient settings may consider treating sleep not as a peripheral symptom but as a core component of crisis prevention and recovery planning. Our data show that caregivers of children with sleep problems, particularly sleep maintenance problems, have more caregiver distress, more dysfunctional caregiver-child relationships, and perceive their child as more difficult to care for than caregivers of children without sleep problems. Addressing sleep maintenance problems during and after hospitalization may strengthen caregiver resilience, reduce stress, and improve the family’s capacity to manage challenging behaviors safely at home.

Generally, children’s sleep problems had a larger detrimental effect on caregiver self-efficacy for single caregivers and those with lower household incomes. These results indicate that child sleep problems may be a family-systems-level issue but depend on family context. Future research should investigate whether systematic inpatient sleep assessment and targeted intervention reduce readmission risk and lead to identifying effective implementation strategies for resource-limited families. Potential solutions may include family or couples therapy, caregiver stress and self-efficacy interventions, financial buffers, and other targeted interventions for caregivers.

## Figures and Tables

**Table 1:** Participant characteristics across sleep problem groups.

	Total (N = 598)	No Sleep Problems (N = 248)	Sleep Initiation Problems (N = 90)	Sleep Maintenance Problems (N = 99)	SI + SM Problems (N = 161)	Significance
						*F*/chi-square

**Age (years) – mean (SD)**	12.9 (3.5)	12.9 (3.5)	12.6 (3.6)	13.7 (3.4)	12.4 (3.5)	**3.2** *
**Sex - male N (%)**	480 (80.3)	200 (80.6)	71 (78.9)	74 (74.7)	135 (83.9)	**3.3**
**Race**^a^ **- white N (%)**	538 (90.0)	225 (90.7)	78 (86.7)	90 (90.9)	145 (91.1)	**1.3**
**Ethnicity**^a^ **– Non-Hispanic N (%)**	547 (91.5)	228 (92.0)	79 (87.8)	91 (91.9)	149 (92.5)	**1.3**
**Caregiver – Biological Parent N (%)**	466 (77.9)	191 (77.0)	75 (83.3)	71 (71.7)	129 (80.1)	**3.8**
**Household Income - > US$50k N (%)**	322 (53.8)	137 (55.2)	42 (46.7)	55 (55.6)	88 (54.7)	**2.2**
**Single Parent Household N (%)**	208 (34.8)	90 (36.3)	30 (30.3)	31 (31.1)	57 (35.4)	**0.9**
**Non-Verbal IQ Score - mean (SD)**	77.0 (27.9)	79.5 (28.0)	78.4 (28.1)	68.7 (24.6)	76.7 (28.9)	**3.0** *
**Profound Intellectual Disability N (%)**	134 (22.4)	49 (19.8)	19 (21.1)	27 (27.3)	39 (24.2)	**5.5**
**Minimally Verbal N (%)**	268 (44.8)	94 (37.9)	40 (44.4)	50 (50.5)	84 (52.2)	**9.6** *
**Profound Autism**^b^ **N (%)**	292 (48.8)	109 (44.0)	42 (46.7)	55 (55.6)	86 (53.4)	**5.7**
**ADOS Comparison Score- mean (SD)**	7.8 (1.6)	8.0 (1.6)	7.9 (1.6)	7.6 (1.4)	7.6 (1.7)	**1.8**
**ABC-Irritability Score- mean (SD)**	26.7 (9.3)	25.8 (9.6)	27.2 (9.0)	27.0 (8.8)	27.6 (9.4)	**1.4**
**Supplemental Melatonin**^c^ **N (%)**	140 (27.9)	48 (23.0)	27 (37.0)	23 (27.1)	42 (31.3)	**6.3**

* p < 0.05.

a Race and ethnicity were self-reported by participants using federally standardized demographic categories established for research reporting.

b Profound autism = co-occurring profound intellectual disability and/or minimal verbal ability.

c Supplemental melatonin use data available for N = 501 participants.

**Table 2:** Multivariate analysis results for caregiver stress and self-efficacy.

	Parental Distress		Dysfunctional Parent-Child Interactions		Perception of Child as Difficult		Difficult Behavior Self-Efficacy	
	Unadjusted	Adjusted	Unadjusted	Adjusted	Unadjusted	Adjusted	Unadjusted	Adjusted

	*β*	*β*	*β*	*β*	*β*	*β*	*β*	*β*
Sleep Initiation	0.03	0.02	−0.01	−0.02	0.08	0.07	−0.05	−0.04
Sleep Maintenance	0.10 *	0.09 *	0.14 ***	0.13 **	0.13 **	0.12 **	−0.08	−0.07
SI + SM	0.11 **	0.10 *	0.08	0.06	0.18 ***	0.15 ***	−0.06	−0.04

* p < 0.05,

** p < 0.01,

*** p < 0.001; effect sizes for all significant effects ranged from negligible to small (*η*^*2*^ = 0.01–0.02).

## Data Availability

Data are available for approved investigators through SFARI Base, maintained by the Simons Foundation Autism Research Initiative.
